# Engaging communities in addressing antimicrobial resistance: Co-producing locally relevant public health messages

**DOI:** 10.1371/journal.pgph.0006212

**Published:** 2026-04-17

**Authors:** Winifred Maduko, Mike Kesby, Jo Mhairi Hale, Emmanuel Olamijuwon

**Affiliations:** 1 School of Geography and Sustainable Development, University of St Andrews, Scotland, United Kingdom; 2 Heroes of Our Time Africa Foundation, Enugu, Nigeria; Institute of Development Studies, UNITED KINGDOM OF GREAT BRITAIN AND NORTHERN IRELAND

## Abstract

Antimicrobial resistance (AMR) is a critical global health challenge that weakens the effective treatment of common infections and threatens health system functioning, particularly in settings where access to affordable healthcare and regulated medicines is limited. This study explores whether grassroots, community-driven approaches can improve the reception of AMR communication materials. Using Arts-Based Participatory Action research methods, 30 community members in Enugu, Nigeria collaborated in creative workshops to co-produce locally relevant AMR messages through posters, jingles, and a short film, delivering messages that promote responsible antibiotic use. These materials were subsequently evaluated through a wider public engagement and exhibition event involving 66 community members, where pre- and post- event surveys assessed community members’ perceptions of the acceptability, cultural appropriateness, and their likely effectiveness in influencing public perception and behaviour. This exploratory study finds that co-produced materials were well received, with participants describing them as culturally embedded, socially acceptable, and potentially effective in raising awareness about antimicrobial resistance. The findings reveal that participatory design of public health communication can enhance message relevance, improve public engagement, and foster community ownership in AMR awareness efforts. However, challenges such as resource constraints, and scalability limitations highlight the complexities of community-driven public health interventions. By employing both qualitative and quantitative methods, the study calls for a shift from conventional expert-led campaigns to inclusive, community-driven strategies in tackling AMR. Curtailing AMR demands public health interventions that are engaging and responsive to the specific social and structural contexts in which antibiotic use occurs.

## Introduction

Antimicrobial resistance (AMR) is a major global health threat, contributing to rising morbidity, mortality, and economic costs [1,2]. In 2019 alone, AMR was directly responsible for about 4.95 million deaths globally with the highest burden of AMR in Sub-Saharan Africa [3]. Although more recent global estimates are still being compiled, recent literature continues to highlight the rising threat of AMR worldwide [1,4]. The overuse and misuse of antibiotics, particularly in Low- and Middle-Income countries (LMICs), have exacerbated resistance, causing many common infections to become increasingly difficult to treat [5,6]. While global and national action plans, including the WHO’s Global Action Plan on AMR, highlight priorities such as public awareness, stewardship, and behaviour change, progress in translating these strategic commitments into effective community-level action remains uneven. In many LMICs, public awareness of AMR remains limited, and where knowledge exists, it does not always translate into appropriate antibiotic-use practices, especially in contexts where antibiotics are readily accessed without prescription [7–10].

A major tension in current AMR responses lies in the disconnect between the recognized complexity of the causes of AMR, which are structural, environmental, and relational, and the often individualized and siloed interventions most commonly promoted. Chandler (2019) [11] highlights how individualized approaches could often fail because they overlook weak health systems, economic precarity, and infrastructural constraints shaping everyday antibiotic use. For example, poor access to healthcare may encourage self-medication, while the informal sale of antibiotics frequently occurs in settings with overstretched health systems. In conjunction with addressing structural issues, public education remains an essential, though not sufficient, component of AMR strategy, ensuring that people understand what antibiotics are, how they work and the implications of their use. In many communities, misconceptions about antibiotics and lack of knowledge persist, partly due to gaps in communication, for example between healthcare providers and the public [12–14]. Participatory approaches could offer a way to enhance such AMR literacy by promoting informed decision-making at the individual level.

The dominant approach to AMR communication remains top-down and expert-led. Messages are often created by scientists, policymakers, or health officials, with limited participation from local communities. Rather than merely disseminating factual knowledge, AMR communication strategies should better reflect the circumstances of the communities they target. This includes recognizing how local values, beliefs, and behaviours shape perceptions of not just antibiotic efficacy, as well as addressing issues of access and representation. Participatory approaches, especially participatory action research (PAR), provide a promising path forward. They allow for co-production of public health materials that are more resonant, practical, and rooted in everyday lives. Addressing AMR effectively requires moving beyond top-down information campaigns to genuinely involving local communities in the production of knowledge and in the design of public health messages, through participatory and culturally grounded forms of engagement. To strengthen AMR interventions and public messages, more holistic, community-led approaches are needed, ones that acknowledge these socio-material realities and actively involve the people most affected.

A growing body of literature highlights that co-production, which forms part of participatory approaches, in health communication can enhance both the effectiveness and equity of public health interventions. Systematic reviews have shown that when communities are actively engaged in co-producing materials, messages are more likely to resonate with local values, be culturally appropriate, and foster a sense of ownership that improves uptake [15,16]. For instance, a study demonstrated how participatory design involving seniors and people with disabilities produced multilingual communication tools that were not only accessible but also trusted [17]. Lessons from past co-creation initiatives highlight several recurring strengths. Participatory processes often surface cultural idioms, metaphors, and storytelling practices that make health risks and recommendations more relatable [18]. For AMR communication, where structural and cultural complexity shapes antibiotic use, theory-informed co-production may be particularly important for ensuring interventions are scalable and sustainable across diverse contexts. At the same time, the literature also points to some important limitations and cautions. Co-production can be resource-intensive and difficult to scale, particularly in resource-constrained contexts [19]. These challenges suggest that while co-production holds promise, its value depends on careful facilitation, attention to equity, and commitment to sustaining participation beyond initial workshops. Overall, these lessons provide a critical backdrop for considering AMR communication.

This study focuses on Nigeria, a country that illustrates broader challenges across Sub-Saharan Africa where the AMR crisis is particularly high. Poor access to water, sanitation, and hygiene (WASH) infrastructure, combined with a high burden of infectious diseases and limited access to affordable healthcare services, increases the problem [4,20]. The informal sale of antibiotics, widespread self-medication practices, and a general lack of public awareness about the dangers of antibiotic misuse further drive the rise of AMR. What all these factors of AMR in Sub-Saharan Africa have in common is that they are mostly deeply rooted in everyday community behaviours, practices, and lived realities. Nigeria bears a substantial AMR burden. In 2019, an estimated 64,500 deaths were directly attributable to AMR, with approximately 263,400 additional deaths associated with resistant infections. Nigeria ranked 185th globally in age-standardised AMR-related mortality, with AMR-attributable deaths exceeding those from tuberculosis, malaria, maternal and neonatal disorders, neglected tropical diseases, and cardiovascular diseases [21,22]. Recent studies continue to identify poor infrastructure, prevalent antibiotic misuse, and limited public awareness as key drivers of resistance [23]. In response, Nigeria has developed a 2024–2028 One Health Antimicrobial Resistance National Action Plan 2.0, which prioritises awareness and behaviour change, surveillance, infection prevention and control, antimicrobial stewardship, and multisectoral governance [21]. The country has also launched its first nationally representative AMR survey and announced plans to reactivate the national antimicrobial stewardship network [24,25]. These awareness efforts have sometimes taken the form of creating messaging materials through posters, health talks, and digital campaigns led or co-ordinated by government agencies, organizations and a range of other stakeholder groups, often framed in biomedical language. These initiatives represent a significant step toward addressing AMR; however, without meaningful community engagement and locally relevant communication interventions, these efforts may not achieve the desired impact. Engaging communities through participatory methods and co-production offers a way to strengthen AMR literacy and community engagement. While this method does not solve infrastructure problems, it is a step toward designing interventions that better motivate behaviour change within existing constraints.

Several studies call for more community-based interventions to address AMR communication and promote appropriate antibiotic use, especially in Sub-Saharan Africa which experiences the highest burden of AMR [26–28]. The need for effective community engagement in AMR communication and community-based intervention is critical, particularly those that move beyond information provision to engage with local meanings, practices and challenges.

This study aimed to explore whether community members, when provided with sufficient education on AMR, can co-produce public health messages that, while evidence-based and scientifically accurate, are also more locally relevant, culturally embedded, inclusive, and actionable for the communities most affected. Specifically, the study addressed three research questions:

1. Given the opportunity to co-produce public health information on AMR and antibiotic use, how do community members frame, represent, and communicate AMR information and antibiotic use?
2. How acceptable and culturally appropriate are the co-produced materials to wider community members?
3. How do community members perceive the potential effectiveness of these materials in influencing awareness and behavioural intentions related to antibiotic use?

By shifting from expert-led to community-driven health communication, this study hopes to contribute to the growing discourse on participatory public health interventions as a strategy for AMR literacy and stewardship.

## Methods

### Ethics statement

Ethical approval for the study was obtained from the Ministry of Health Research Ethics Committee in Enugu, Nigeria (MH/MSD/REC21/617), and the School of Geography & Sustainable Development Ethics Committee at the University of St Andrews. All participants in the study provided informed written consent and received an information sheet detailing the study objectives and their participation. After going through the participant information sheet in local language, written informed consent was taken from participants by the study team. The individual pictured in Figs 2–4 has provided written informed consent (as outlined in PLOS consent form) to publish their image alongside the manuscript.

**Fig 1:**
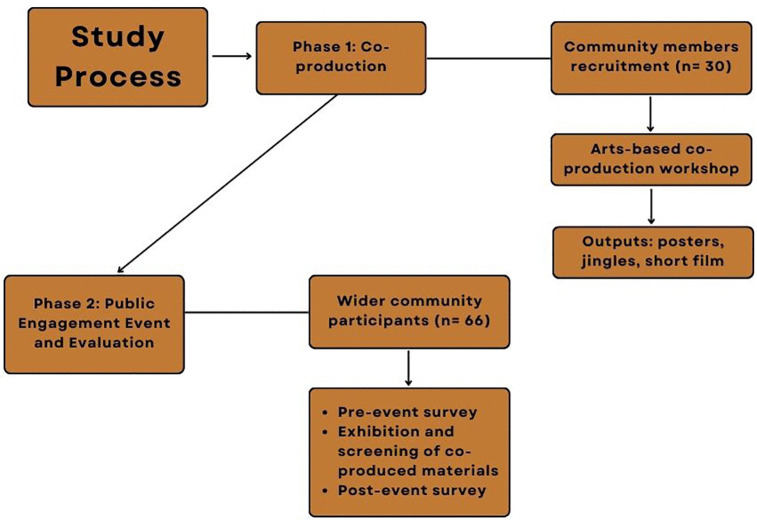
Study Design Process.

**Fig 2:**
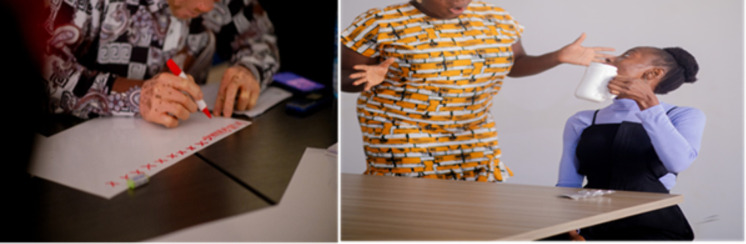
Participants during the co-production creative process.

**Fig 3:**
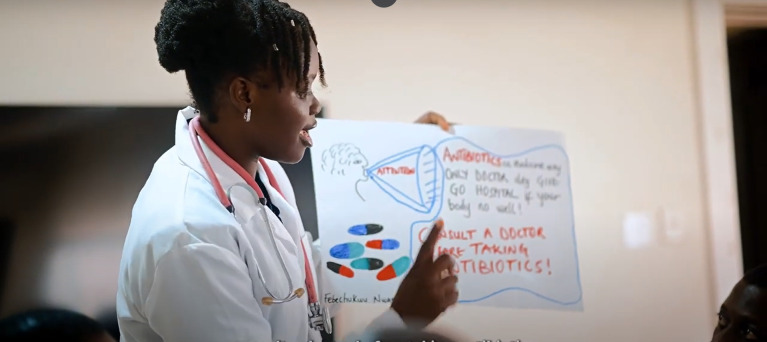
Participants during the community short film production, link to film: https://youtu.be/WA0OV38iebU?si=el5za9pLAoZkv0qj.

**Fig 4:**
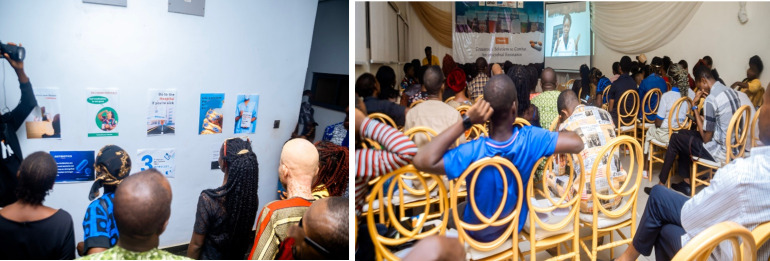
Wider public engagement event and exhibition of co-produced materials.

### Study setting and community context

This study was conducted in Enugu State located in south-eastern Nigeria. Enugu is characterized by diverse socio-economic conditions, including low-, middle-, and higher-income areas. It has a mix of both rural and urban areas across 17 local government areas and has an estimated population of approximately 4 million people [29]. The population is largely Igbo-speaking, with English and Nigerian Pidgin commonly used in everyday communication. Educational attainment varies widely, and livelihoods include civil service, informal trading, artisanal work, and entrepreneurship. Healthcare access in Enugu reflects broader patterns across Nigeria [30]. Public and private health facilities are present, but access to affordable and timely care remains uneven. Many residents rely on patent and proprietary medicine vendors and informal drug sellers for first-line treatment, contributing to widespread self-medication and non-prescription antibiotic use. Previous studies have highlighted limited public awareness of antimicrobial resistance across Nigeria, alongside high rates of inappropriate antibiotic use and weak regulatory enforcement of prescription-only policies [9,20].

### Study design

This study engaged community members in Enugu, Nigeria, in the co-production of public health messaging materials focused on AMR and antibiotic stewardship. It involved two interlinked phases (described below and see Fig 1) and data were collected between July and October 2024. All participants in the study provided informed written consent and received an information sheet detailing the study objectives and their participation. The study built upon methods pioneered by Kesby and Fredricks [31] in their ongoing work in Tanzania and employed participatory action research approaches [32–34] to engage community members as co-creators (rather than passive recipients) of public health information about AMR. The approach begins with assessment of participants’ existing knowledge, attitudes and practices around health-seeking and antibiotic use. Participants were invited to review and critique existing national and international AMR education materials and discuss why they communicated or failed to communicate AMR related knowledge to local populations. Through a series of workshops, participants then discussed the drivers of contextually specific health-seeking as well as the motivations and messages that might promote behaviour change. Then, following training in the use of arts-based methods (video, photography and poster making), participants co-constructed alternative AMR public health information materials they believed would better communicate information to their peers. This study extended Kesby and Fredricks’ approach particularly through the inclusion of a public community evaluation of educational materials produced by the research participants.

The study draws conceptual inspiration from primarily the Health Belief Model (HBM) [35,36] that seeks to explain health behaviours by focusing on people’s perceptions of ill-health, its severity, their own susceptibility, and the perceived benefits of and barriers to behaviour change. From this, the study draws the notion of ‘cues to action’ (things that prompt behaviour change) and ‘self-efficacy’ (patient’s sense they can pursue behaviour change effectively). Although the HBM is limited by an overly individuated approach [37], it remains useful as a sensitizing framework for thinking about how public health messages are framed and received. Bringing these insights together with PAR approaches we explored whether more effective ‘cues to action’ could be co-produced with the community participants who are the targets of public health information. Importantly, because they are more attuned to local norms, values and contexts than top-down information designed by external experts. Further, we explored if ‘self-efficacy’ would be increased because of the sense of collective ownership of such materials.

Using PAR approaches, we created a collective space in which to explore key elements of the HBM, enabling participants to interrogate how their personal practices around antibiotic use emerge from structural constraints such as insufficiently regulated drug markets, strained health facilities, and community-level norms around self-medication. ‘Perceived susceptibility’ to AMR and ‘perceived barriers’ to efficacious action were explored via group discussion, storytelling and dramatization of community norms, values and relational behaviour/decision-making. Having altered perceptions about the benefits of behaviour change through AMR education sessions, participants then develop co-produced public health messages that, whilst reflecting biomedical accuracy, also incorporated cues to action which mobilised collective understandings and motivations rooted in everyday community experiences.

**Phase 1: Co-Production Workshop** – 30 community members participated in an arts-based participatory workshop to develop culturally relevant AMR public health messages. The workshop began by encouraging participants to review and take note of perceived issues with the existing materials being reviewed and their suggestions (in a previous study, we asked participants to source AMR public health messages). They also raised several fundamental considerations for AMR and antibiotic public health messaging, such as who delivers the message, the intended audience, and the mode of delivery. These insights informed their approach to creating ‘improved’ community led AMR and antibiotic stewardship messages during the creative section. Participants in this discussion and creative co-production workshop collaboratively co-created a range of communication materials, including posters, jingles, and short films, tailored to reflect local language, values, and practices. The co-production process allowed for strong participant input in design and creation, with the researcher playing a facilitating role. During the workshop, participants reviewed some retrieved existing local, national, and international public health messaging materials on AMR and antibiotic use, including posters, audio, video, and online campaigns spanning from 2019 to 2024. The primary target audience for the co-produced materials were community members who commonly encounter antibiotics in their everyday life, including informal users, caregivers, family decision-makers, etc. While secondary audiences included the wider community, local media, and community institutions involved in health information dissemination.

**Phase 2: Public Engagement Event and Evaluation** – A wider public exhibition and feedback session was organized, drawing 66 participants from the community, excluding the initial 30 co-producers. The event, which lasted approximately three hours, was held at a centrally located community event centre to ensure accessibility. During this event, the co-produced materials were exhibited. The event featured screenings of the short film, playing of jingles and displays of posters, together with educational health talks on AMR and interactive sessions. Participants’ perception of their potential impact and level of acceptability was assessed using structured pre- and post-intervention surveys and the presentation of the coproduced materials. Feedback was gathered through the pre- and post-surveys to evaluate community members’ perceptions of the acceptability, clarity, and impact of the new materials. Traditional media representatives (e.g., radio and television) were present at the event, which ensured that the public health information highlighted at the event were disseminated beyond the 66 participants who attended in person.

### Workshop design and facilitation

The co-production workshop was designed as a structured but flexible process that combined education, collaborative critique, and creative production. The first session introduced participants to foundational knowledge of AMR and antibiotic stewardship using short talks, simple visuals projected on a screen, and interactive Q&A to establish a common baseline of understanding. Participants then engaged in a review of existing local, national, and international AMR communication materials such as posters and drawings. They were encouraged to identify aspects they found confusing, unrelatable, or (in)effective, as well as features that could be improved to resonate with their community. The creative phase involved small-group discussions where participants brainstormed ideas for more effective messages, drawing on their lived experiences, languages, and cultural expressions. Arts-based methods were central to the workshop. Tools such as storyboarding, role-play, drawing on flipcharts, and song composition, were used to spark creativity and ensure contributions from participants with varying literacy levels. Participants used A3 sheets, markers, and stickers to design poster drafts, role-played short scenarios that were later adapted into a filmed script, and engaged in collaborative songwriting and script development for jingles in English, Igbo, and Pidgin. These methods encouraged experimentation and iterative revision processes. For example, there were some groups that debated language choices before finalizing slogans or adapted familiar proverbs into the jingles. The study emphasis was on creativity, cultural resonance, and inclusivity. As researchers, we adopted a participatory role, supporting rather than directing, ensuring all voices, including those of persons with disabilities, were equally heard. The final stage of the workshop focused on collaborative synthesis, groups presented their initial designs and ideas for the posters, scripts, and jingles to the larger group, which collectively refined them into final outputs.

### Participant recruitment

Participants for the co-production workshop were recruited with the support of South-Saharan Social Development Organisation (SSDO - a non-profit organisation in Nigeria). Initial potential participants were identified, and they then helped recruit others through a snowball approach. While the sample was not structured to be statistically representative or to prioritise specific subgroups (e.g., high antibiotic users or caregivers), efforts were made to ensure diversity in gender, age, and lived experience, including the participation of persons with disabilities. A total of 30 community members, representing women and men and including persons living with disabilities (PLWDs), were recruited for the co-production workshop. The inclusion of persons with disabilities was deliberate, to address a critical gap in AMR intervention research within Sub-Saharan Africa. People with disabilities (PLWDs) often face heightened vulnerability to infectious diseases and AMR due to limited access to healthcare, educational barriers, and, potentially, higher rates of comorbidities [26]. Including persons with disabilities is therefore essential to ensure interventions account for their unique needs and perspectives of PLWDs. By involving them in co-production, we aimed to create public health messages that resonate across diverse population segments. All 30 co-production participants were briefed on the project’s objectives and provided with options regarding their level of participation (e.g., full identifiable participation, anonymous contribution, or behind-the-scenes involvement).

Participants for the wider public engagement event were recruited using a community-based and relational approach that leveraged existing networks and local partnerships, with recruitment facilitated through local contacts who shared information about the event within their communities. In addition, the non-governmental organization (SSDO) supported outreach efforts by helping to identify and invite interested individuals. Participation was voluntary and not intended to be statistically representative. Potential participants were first contacted and provided with a brief explanation of the event’s purpose (to showcase and gather feedback on co-produced public health messages addressing antimicrobial resistance and antibiotic stewardship). This approach helped manage attendance numbers and control audience size while ensuring that the event remained inclusive and accessible to a diverse cross-section of the community. This approach to recruitment reflected the study’s broader commitment to grassroots engagement and co-production, ensuring that participants were drawn from the same communities whose perspectives had informed the creation of the public health materials.

### Development of the questionnaire

The public engagement pre- and post-surveys used to evaluate the acceptability and perceived effectiveness of the co-produced AMR public health materials were developed specifically for this study. The design of the questionnaires used was informed by a review of existing literature on public health communication, antimicrobial resistance awareness, and community-based participatory research. Questions for the pre- and post-intervention surveys were also shaped by insights from earlier phases of the project, particularly around participants’ understanding, beliefs, and practices related to antibiotic use. The surveys included both closed-ended and open-ended questions to allow for quantitative analysis as well as richer qualitative feedback. Key themes covered included demographic characteristics, baseline knowledge and awareness of AMR, exposure to AMR messaging (pre-intervention), perceptions of cultural relevance, and intentions to adopt improved practices following the intervention.

The pre-survey consisted of four sections (S1 Text). The first section collected demographic and contextual information, including age, gender, educational level, access to media and communication platforms, recent antibiotic use, frequency of antibiotic use, and whether participants had previously obtained antibiotics for themselves or others without medical advice. The second section focused on awareness of antimicrobial resistance, asking participants how aware they were of AMR, whether they had previously seen or heard public health messages about AMR or antibiotic use, where such messages were encountered, and how likely they believed people in their community would be to pay attention to AMR-related public health messaging. The third section explored participants’ initial perceptions of public AMR communication prior to exposure to the co-produced materials, including the perceived importance of educating the public about AMR and responsible antibiotic use. The final section captured expectations regarding the effectiveness of different forms of public health communication, asking participants whether they believed messages produced by experts, by the public, or through co-production were most effective. Participants were also asked about what makes public health campaigns effective in their view and how effective they expected the co-produced materials (posters, jingles, and short film) to be in raising AMR awareness.

The post-survey followed a similar structure but focused on participants’ reflections after engaging with the co-produced materials during the exhibition event (Supporting Information 1). It comprised three sections. The first assessed perceived effectiveness, asking participants how effective they felt the materials were in raising awareness of AMR, which formats they found most impactful, and which media channels would be most appropriate for dissemination within their community. The second section focused on acceptability and cultural relevance, exploring participants’ agreement with statements regarding the cultural appropriateness of the materials, comfort with language, imagery, and presentation style, and reasons underlying these assessments. The final section examined behavioural intentions and engagement, asking participants how likely they were to take specific actions to prevent AMR (e.g., responsible antibiotic use, sharing information with others), whether they would recommend the materials for use in other communities, what they found most useful, and how the materials could be improved for greater effectiveness. While pre- and post-surveys were administered to the same participants and could be linked at the individual level, the primary aim of the evaluation was not to conduct formal pre-post explicit comparisons. Rather, the surveys were used to explore participants’ perceptions of acceptability, cultural relevance, and perceived effectiveness of the co-produced materials following the exhibition.

### Validity and pretesting of the questionnaire

Content validity of the questionnaire was ensured through a detailed review by the research team to confirm clarity, relevance, and coverage of key thematic areas. Face validity was assessed through an informal pretest with the local NGO partners in Enugu, Nigeria to ensure questions were understandable, culturally appropriate and contextually relevant. Necessary adjustments were made following their feedback from the pretesting process, for example revisions were made to question wording and terminology to improve clarity.

### Data analysis

Descriptive statistics, including frequency counts and percentages, were used to summarise responses to closed-ended questions and are presented in tables to aid interpretation (Microsoft Excel). Responses to open-ended questions were analysed qualitatively using an inductive thematic approach, guided by an interpretivist epistemological perspective consistent with participatory action research [34,38,39]. This approach assumes that meanings are socially constructed and situated, and prioritises participants lived experiences, language, and locally grounded interpretations rather than imposing predefined analytic categories. Qualitative responses were reviewed to identify key themes relating to clarity, cultural relevance, acceptability, and perceived impact of the co-produced materials. These qualitative responses were integrated with the quantitative findings to provide contextual depth and enhance interpretation of the findings. The qualitative analysis was first conducted by WM, with review and discussion on the drafts involving the wider research team. Analysis proceeded in several stages. First, all qualitative materials were read repeatedly to achieve familiarisation. Initial codes were then generated inductively, without applying a pre-existing coding framework, allowing themes to emerge from participants’ own language and expressions as captured in the survey. These codes were iteratively reviewed and grouped into broader themes capturing key patterns related to acceptability, cultural relevance, and perceived effectiveness. Participants were not involved in checking or refining themes. Data informing the reflections on the workshop process presented in the results section below were drawn from audio recordings of the co-production workshop discussions, including plenary conversations and small-group activities. The workshop sessions were audio-recorded with participants’ consent and subsequently transcribed. Where necessary, transcripts were translated into English by WM. The transcripts were reviewed, and the data is descriptively reported. No separate individual interviews were conducted; reflections were generated from the participatory activities.

### Inclusivity in global research

Additional information regarding the ethical, cultural, and scientific considerations specific to inclusivity in global research is included in the Supporting Information (S1 Checklist).

### Reflexivity and positionality statement

The lead author (WM) is a Nigerian researcher and PhD candidate with prior professional experience in community development, public engagement, communication and advocacy in Nigeria. This positionality shaped both access to the field and the design of the participatory process alongside the contributions and supervision of the co-authors (MK, EO, JH), who bring extensive experience in AMR projects and health research in contexts such as Nigeria, Tanzania and Uganda. Familiarity with local languages, cultural norms, and everyday challenges surrounding healthcare access in Nigeria particularly in Enugu, facilitated trust-building with participants and supported the use of culturally grounded, arts-based methods during the workshop and public engagement event.

## Results

### Co-production workshop outcomes

Given the focus on creating engaging, locally relevant public health messages on AMR and antibiotic stewardship, workshop participants co-produced engaging posters (which they featured in and creatively designed), audio jingles (in different local languages using local idioms and expressions) and an awareness community-based short film (see Table 1 **below for participant demographics and** S1 Table). By centring on familiar visual and linguistic elements, the new messages aimed to bridge gaps identified in existing AMR-related communications. During the creative session participants used A3 poster cards and markers to express their ideas and preferences for the co-produced materials before producing the final graphic designs. Fig 2 captures the participants during the co-production creative process. The participants collaborated in groups to generate action posters, a short community-based awareness film (See Fig 3 **and the link to the video**) and three audio jingles (in Igbo, English and Pidigin languages). Importantly, the co-production process highlighted how a participatory lens can expand beyond the individualistic implications of HBM. For example, while the HBM might suggest framing messages around “personal risk” or “individual benefits of completing a dosage,” participants in some of their designs re-framed these messages in collective and relational terms. One group designed a poster emphasising that “misusing and overusing antibiotics puts us all at risk”, directly linking individual actions to community wellbeing. Similarly, the short film situated antibiotic misuse within strained health systems and economic hardship, reflecting structural barriers often neglected in individual-behaviour models. These outputs highlight how participatory approaches enabled participants to embed AMR awareness into shared cultural idioms and collective responsibility.

**Table 1:** Demographic characteristics distribution of co-production workshop participants (n = 30), PWDs: Persons with disabilities.

Category	Subcategory	Number (n)	Percentage (%)
Age Group	18-30	6	20%
	31-50	21	70%
	51+	3	10%
Gender	Male	14	47%
	Female	16	53%
Education Level	No formal education	2	7%
	Primary	5	17%
	Secondary	8	27%
	Tertiary	13	43%
	Tertiary (Still in school)	2	7%
PWDs	PWDs	5	17%

### Reflections on the workshop process

Insights emerged from the workshop process itself, beyond the tangible outputs. First, participants emphasized that being invited to critique existing AMR materials validated their lived experiences and positioned them as knowledge-holders rather than passive recipients. This early stage of critical review-built confidence and created a sense of ownership over the subsequent creative process. Second, the arts-based methods proved vital in facilitating inclusion. Role-play and participating in the drama (for the short film) helped some participants with limited literacy contribute equally, while drawing and local proverbs provided culturally- rooted expressions of AMR risks. Persons with disabilities highlighted how multimodal formats (visuals, audio, and storytelling) improved accessibility –an insight that directly shaped the final designs. Third, the process generated unexpected peer learning. Participants debated appropriate metaphors (e.g., whether to frame antibiotics as “helpers” or “dangerous friends”), which led to richer outputs and more collective reflection. These conversations also revealed structural barriers, such as the difficulty of seeing a doctor in contexts where access is limited. Participants acknowledged these constraints; nonetheless, they wanted messages to encourage responsible use while also expressing among themselves the need for broader systemic reform. Importantly, the workshop process itself became an intervention, participants described feeling empowered to act as advocates even before the materials were formally disseminated. Some of the participants reported discussing antibiotic misuse with family members after the first sessions we had. This suggests that co-production can simultaneously produce effective communication tools and could improve behaviour change by activating participants as peer educators, which is exactly what we intend to build further with this study (peer to peer learning and engagement). During the public engagement exhibition and evaluation phase of this study, some of the co-production participants were invited as resource persons sharing their experiences and learning to wider community members at the event. Overall, the co-production workshop helped in generating different AMR messaging materials and it was a good learning opportunity for the participants.

The inclusion of persons with disabilities (PWDs) in the co-production workshop had a tangible influence on both the design process and the final AMR co-produced outputs. Their participation prompted early and sustained attention to accessibility, especially regarding assumptions about how health messages are typically communicated. During group discussions, PWD participants highlighted the need for co-produced materials to consider people with visual, hearing, or cognitive impairments. This directly informed the group’s collective decision to prioritise the co-production of multimodal formats, including audio jingles, visual storytelling, and dramatization through film. PWD participants also shaped how inclusivity was conceptualised beyond disability-specific needs. For example, discussions about clarity, pacing, and simplicity of the co-produced messages and materials. Importantly, PWDs contributed substantive content insights based on their lived experiences. Some participants drew attention to frequent interactions with healthcare systems, higher exposure to antibiotics due to chronic conditions, and difficulties accessing good medical support. These perspectives influenced how risk and responsibility were framed in the messages used in the co-produced materials. Additionally, the insights that emerge from PWDs are not only useful and relevant to PWDs, but also to the population more broadly. Thus, the use of multiple, multi-sensory formats to disseminate the same message is beneficial not only for people with visual or hearing impairments, but for the wider community as well.

### Public engagement event evaluation

The final co-produced materials were strategically displayed in an exhibition format during the wider public engagement event to evaluate the acceptability of the co-produced messages interventions by other community members (see Fig 4). Sixty-six community members participated in the public engagement event and awareness lecture. The sections below show the results of the pre-and post-survey.

### Participant demographics, practices, and AMR awareness

A total of 50 participants completed the full survey (see Table 2 **below for participant demographics, antibiotic-use practices, and selected indicators. Summary of survey response distributions are provided in** S2 Table). They represented a wide age range, with the largest group aged 18–25. Education levels were relatively high, with nearly 60% having tertiary education. Technology access was notable, over three-quarters owned smartphones and used social media, suggesting a strong potential for digital dissemination of AMR messages, alongside traditional media platforms. Despite this, awareness of antimicrobial resistance was limited before the event, more than half had never encountered any AMR messages. Most participants reported prior misuse of antibiotics, including self-medication and recent unsupervised use. Encouragingly, 86% believed their communities would pay attention to AMR messaging, suggesting high receptiveness to better-informed public health communication.

**Table 2:** Summary of Public Engagement Event and Evaluation Participant Demographics, Practices, and selected indicators.

Category	Subcategory	Number (n)	Percentage (%)
Age Group	18-25	18	36%
	26-35	11	22%
	36-45	9	18%
	46-55	6	12%
	56+	6	12%
Gender	Male	24	48%
	Female	26	52%
Education Level	Primary	1	2%
	Secondary	19	38%
	Tertiary	30	60%
Access to Communication technologies	Access to radio and TV	26	52%
	Use social media	38	76%
	Own smartphone	39	78%
Antibiotic Use and Practices	Used antibiotics in last week/month	11	22%
	Used antibiotics in last 6 months	14	28%
	Used antibiotics occasionally	15	30%
	Took antibiotics without medical advice	33	66%
AMR Awareness	Very aware	14	28%
	Not at all aware	13	26%
	Had not seen/heard AMR messages before	29	58%
Likely to Pay Attention to AMR Messaging	Likely/Very likely	43	86%

### Initial perceptions and expectations of AMR messaging

Before seeing the co-produced materials, a strong majority (86%, n = 43) agreed that educating the public about AMR and responsible antibiotic use was “very important.” When asked, through a closed multiple-choice question, about the most effective type of public health communication, 62% (n = 31) believed co-produced messages, developed collaboratively by experts and the public, were more impactful than those created solely by professionals (30%, n = 15) or the public (6%, n = 3). Expectations for the co-produced materials were high: 42% (n = 21) expected them to be “very effective,” and 30% (n = 15) anticipated they would be “extremely effective.” Additionally, about 91% (n = 46) of participants agreed or strongly agreed that community involvement in creating health messages increases their effectiveness. This was also measured through a closed Likert-scale question. Open-ended responses highlighted the importance of media channels (radio, social media, short films), community involvement, and practical relevance for campaign success.

### Perceived effectiveness of the co-produced materials (Post Intervention Survey)

After engaging with the co-produced posters, jingles, and short film, 92% (n = 46) of participants rated them as either “extremely effective” (46%, n = 23) or “very effective” (46%, n = 23) in raising awareness about AMR. Notably, 60% (n = 30) of respondents found all the materials equally impactful, while 22% (n = 11) favoured the short film and 10% (n = 5) selected jingles. Explanations for these preferences, participants indicated that the materials worked well because they combined multiple modes of communication, were relatable and memorable, and used local language. For example, a participant noted, “they [all co-produced materials] were all equally impactful because whether you are dumb, deaf or blind you still have a chance to be informed”. When asked how best to disseminate the materials, social media was the top choice (30 mentions), followed by radio (16) and TV (15).

### Cultural relevance and engagement

Cultural acceptability of the materials was rated highly, with 88% (n = 44) of participants agreeing or strongly agreeing that the outputs were “culturally appropriate”. This term was presented in a Likert scale question; however, participants also elaborated by giving reasons on their understanding in follow-up open-ended questions. Participants reasons included the use of local language and relatable scenarios, sensitivity to cultural beliefs, and clear, inclusive messaging. They appreciated the use of local languages, relatable content, and accessible formats in response to the questionnaire prompts. Similarly, 96% (n = 48) agreed or strongly agreed that involving community members improved the materials’ effectiveness. Additionally, 86% (n = 43) of respondents felt “very” or “extremely” comfortable with the language, imagery, and presentation style. Only one participant reported slight discomfort.

### Behavioural intentions and future actions

After the public engagement session, 92% (n = 46) of participants expressed a high likelihood (either “very likely” or “extremely likely”) to take specific actions to prevent AMR. Common actions included spreading awareness (19 mentions), educating others (10), and using antibiotics responsibly (9). Participants overwhelmingly (82%, n = 41) would “definitely” recommend the co-produced materials for use in other communities, with an additional 18% (n = 9) responding “probably yes.”

### Suggestions for improvement

When asked how the co-produced materials could be improved, specific suggestions for improving the materials focused on wider dissemination, especially to other rural areas, and using social media, schools, religious gatherings, and public places like restaurants and markets. Integration into schools and translation into other local dialects and continuous sensitization efforts.

## Discussion

This study adds important findings to the literature on participatory approaches in global health strategies. It demonstrates the potential of involving communities in the co-production of culturally relevant public health messages on antimicrobial resistance (AMR). By highlighting community voices and embedding local realities in message design, the study not only produced communication tools but also fostered ownership and understanding among participants. This sense of ownership was particularly evident in the way participants engaged in the process of co-producing AMR messages in this study. Moreover, the perceived high engagement levels, positive evaluations of the materials, and intention to act post-intervention during the survey, affirm the potential value of participatory, context-specific approaches in addressing complex public health issues like AMR. While the findings indicate high levels of acceptability, cultural resonance, and reported intentions to act, this study was not designed to assess actual long-term outcomes and behavioural change. The evaluation relied on self-reported perceptions collected immediately following exposure to the co-produced materials and should therefore be interpreted as exploratory and indicative rather than causal. The study is best understood as a proof-of-concept demonstrating the feasibility and perceived value of community co-production in AMR communication, rather than a definitive assessment of intervention effectiveness.

In relation to the first research question of this study, which is how community members frame, represent, and communicate AMR information and antibiotic use, the co-production process, involving 30 diverse community members, proved critical to crafting materials that resonated with local audiences. Unlike some traditional expert-led campaigns, these messages emerged from discussions about existing AMR messaging materials, lived realities, language, cultural beliefs, and perceived barriers to antibiotic stewardship. Participants, for instance, pointed out that some existing messaging materials sometimes used unfamiliar scientific terms and sometimes lacked the emotional or cultural cues needed to connect with everyday experiences. In contrast, the co-produced posters, jingles, and the short film used local idioms, storytelling, and real-life scenarios, deliberate choices that amplified relatability and engagement. While some international agencies may avoid such culturally specific elements to ensure global applicability, this approach can be applicable in national and sub-national campaigns, where cultural adaptation is both possible and essential. This aligns with participatory literature that highlights how meaningful involvement in health message design enhances the perceived relevance and effectiveness of communication interventions [17–19]. Importantly, this study contributes further by showing that co-production does not just improve message design, it also empowers participants as agents of change within their own communities. More critically, it gives them a sense of co-ownership and some control over the information they receive and share. The study supports existing literature advocating for participatory approaches in health communication [16,40].

Co-producing messages with community members ensures that information is framed in ways that resonate with the target audience, enhancing comprehension and behavioural impact. The quality of the messages co-produced (see supporting information 4), was generally high, with most messages clearly encouraging responsible antibiotic use and advising people to consult health professionals. However, this raises important questions about feasibility. While advising people to “see a doctor” aligns with medical guidelines, in many Nigerian communities, this advice may not be practical due to economic constraints, overburdened health systems, and limited access to affordable care. As Chandler (2019) [11] and others have pointed out, the overemphasis on individual responsibility in AMR messaging can obscure these structural barriers. Participants’ responses also revealed important boundary conditions for the impact of AMR literacy interventions. Feedback by participants pointed to systemic barriers that co-produced materials alone may not overcome AMR. For instance, participants acknowledged that knowledge and awareness would be insufficient without access to affordable healthcare and broader health system reforms. There is need for AMR interventions to be situated within broader efforts to strengthen health systems, including ensuring access to regulated pharmacies, universal health coverage, and enforcement of prescription-only medicine policies. Post-event findings showed strong endorsement of the co-produced materials’ effectiveness. Although responses were overwhelmingly positive, it is important to acknowledge that the evaluation immediately followed the intervention, and participants may have felt compelled to respond positively due to social desirability or the nature of the questions asked. Participants found the short film particularly impactful due to its dramatized content, local language, and visual storytelling. Others favoured jingles and posters for their portability and memorability. A key finding was that 60% of participants found all formats equally impactful, suggesting that using multi-modal channels can maximize reach and accommodate varied learning preferences.

These findings suggest that participatory approaches could complement the Health Belief Model by thinking about the key elements of the model, and the drivers of individual decision-making in more social, contextual and relational terms. While HBM identifies perceived susceptibility, severity, benefits, and barriers as determinants of behaviour [36], our study highlights insight into how ideas related to susceptibility, severity, benefits, and barriers were framed in collective and relational ways during the participatory message co-production. For instance, participants highlighted their perceived barriers to health system constraints such as the affordability of seeing a doctor, while cues to action were reimagined as communal responsibilities expressed through the design in local proverbs and shared storytelling. This further demonstrates how participatory practices could foster communication that acknowledges the socio-economic realities in which antibiotic use occurs. In so doing, the intervention highlights how HBM can be adapted through participatory design to generate more socially embedded and culturally resonant public health messaging.

Cultural appropriateness emerged as a central strength of the intervention. Respondents appreciated that materials reflected their environment, values, and language. Elements such as the use of Pidgin English and Igbo, inclusion of traditional music and references, and portrayals of common healthcare behaviours increased emotional resonance. As one participant stated, the materials were relatable “whether you are dumb, deaf, or blind, you still have a chance to be informed.” Such variety of information design, enabled by co-production, broadens the reach of AMR campaigns, especially among marginalized groups like persons with disabilities. Also, designing messages that are accessible to persons with disabilities also improves clarity and utility for the wider population, demonstrating that inclusive communication enhances overall communication quality rather than benefiting only the PWDs group.

In addressing the second research question, 96% of respondents believed that community involvement improved the co-produced materials perceived effectiveness, highlighting the idea that health messaging may be more persuasive when it emerges from the people it aims to serve. This finding is in line with the key tenets of the Health Belief Model such as perceived benefits and self-efficacy, which emphasizes that people are more likely to engage in health-related behaviour when they perceive messages as relevant to their own circumstances and when they are involved in shaping those messages [36]. However, since this exploratory study did not track actual behaviour change; these results should be interpreted as preliminary indicators. Future work should assess behavioural outcomes and scalability.

One of the most promising findings of this study, which is in relation to the third research question, is that 92% of participants indicated they were likely or extremely likely to take action to prevent AMR, following exposure to the co-produced materials. Specific intended actions included educating family and neighbours, advocating through religious and social gatherings, and changing personal antibiotic use practices. While these self-reported intentions are encouraging, it is also possible that participants responded in ways they believed were expected of them. These reflections show early indicators of social diffusion, where individual change may influence wider community norms [41]. Furthermore, open-ended responses revealed that participants saw themselves not just as beneficiaries but as advocates, many expressed desires to “take awareness to the grassroots,” “share on WhatsApp,” or “speak to my family about what we have been doing wrongly.” Such perceived narratives demonstrate how co-production followed by interactive community engagement events, could foster a sense of collective responsibility and activate participants as peer educators or champions, further enhancing the sustainability of the intervention. Although reported intentions to change behaviour and share messages by participants were high, these findings should be interpreted cautiously. These responses may reflect social desirability bias or the immediacy of the intervention context.

Suggestions by participants for improvement of the co-produced materials included wider dissemination, particularly in rural areas, and integration into schools, churches, markets, and social media platforms. Several participants proposed translation into more local dialects and inclusion of AMR messaging in school curricula. These ideas show the importance of community-driven dissemination strategies and the need to tailor communication formats to diverse local contexts.

Overall, this exploratory, proof-of-concept study contributes to the growing evidence base advocating for grassroots interventions in global health, especially in the context of AMR in Sub-Saharan Africa. Its use of arts-based participatory methods adds innovation to conventional health communication strategies and demonstrates how creative engagement may bridge biomedical information and cultural meaning-making. By aligning public health messaging with the lived realities of target populations, the intervention achieved a rare combination of relevance, impact, and inclusivity.

Moreover, the study is one of the few to actively include persons with disabilities (PWDs) in the co-design of AMR messages, a demographic often excluded from such initiatives. Their inclusion not only enriched the design process but also expanded the accessibility of the outputs. This is an important step toward achieving equitable public health communication. PWDs contributed valuable insights drawn from their lived experiences during the co-production workshop, particularly regarding their frequent interactions with healthcare systems and increased susceptibility to infections. Research shows that PWDs often have higher rates of comorbidities and may use antibiotics more frequently due to chronic conditions, surgery-related infections, or immune suppression [42]. This places them at heightened risk of exposure to AMR and highlights the importance of tailoring messages to this group. Additionally, the inclusion of PWDs contributed to a deeper sense of community ownership and equity. All members of the society regardless of ability, have valuable knowledge and can contribute to AMR awareness. This aligns with the broader public health call for inclusive communication strategies that leaves no one behind. Moreover, including PWDs not only means that they can give valuable insights, but it also transforms AMR campaign itself into a platform that advances disability inclusion. Given the scale and reach of AMR interventions globally, incorporating PWDs ensures that these moments are not missed opportunities but instead become vehicles for broader social change. Much like how HIV interventions have been used to simultaneously promote gender equity or youth empowerment, AMR-focused efforts can and should serve a dual purpose: tackling resistance while also driving inclusive public health practices.

## Limitations

One limitation of this study concerns the use of fear appeals in some co-produced materials. One poster, for example, warned that “abuse of antibiotics can lead to your doom.” While the participants explained that this language was meant to shock and grab attention, public health research has repeatedly shown that fear-based messaging such as in campaigns on HIV, tobacco, or teenage pregnancy, often fails to produce sustained behaviour change and can even backfire, especially when not accompanied by actionable solutions [43,44]. Furthermore, the use of the word “doom” itself may carry ambiguous or religious connotations and may not clearly convey the health consequences intended. Future co-production processes should consider these risks and balance emotional appeal with evidence-based framing. The final co-produced short film, while created through a participatory process, ultimately adopted a fairly conventional medical narrative structure, where an expert explains risks to community members. This re-authorisation of expert authority while good, may limit the transformative potential of community co-production. It also raises questions about whether participants felt empowered to reimagine alternative formats or simply reproduced dominant communication tropes. Notwithstanding, future work could encourage participants to explore and adopt more community-led and peer-to-peer communication formats. Social desirability bias is another important limitation of this study. The evaluation of the co-produced materials relied on self-reported perceptions and intentions collected immediately after exposure during the public engagement event. Participants may have felt inclined to provide positive responses that aligned with perceived expectations or social norms. This immediate post-exposure timing may therefore have amplified favourable assessments of acceptability, cultural relevance, and intended behaviour change.

Another limitation of this study relates to participant characteristics. A relatively high proportion of the respondents had tertiary education, which may limit the generalisability of findings to populations with lower literacy or educational attainment. This likely reflects who was available and willing to participate in a time-intensive public engagement event, rather than deliberate exclusion. While arts-based and multimodal formats were used to enhance accessibility of the co-produced materials, Future studies should explore how similar co-production approaches perform in settings with lower average educational attainment. A final limitation of the study is that it was conducted in a single location (Enugu, Nigeria), which may affect generalizability. However, this does not suggest that communities elsewhere cannot produce similarly impactful messages (see example Kesby and Fredricks, 2022) [31]. Rather, the point is that while the study provides promising preliminary evidence that community co-production could enhance the perceived acceptability, cultural relevance, and potential effectiveness of AMR communication materials in this specific context, variations in cultural, linguistic, or structural conditions across other regions may influence how such approaches are implemented or received. Future research should explore co-production in multiple communities and how similar participatory strategies perform in diverse geographic, linguistic, and social contexts, including rural and urban settings. Additionally, follow-up studies could assess whether the reported intentions translate into sustained behaviour change over time. Overall, this study demonstrates how community-driven interventions using participatory and arts-based methods can meaningfully contribute to AMR awareness and antibiotic stewardship.

## Conclusion

This study highlights the potential of community co-production in public health communication, particularly in addressing antimicrobial resistance. By engaging communities in the design and delivery of culturally resonant materials (posters, audio jingles, and a short film), this study found that co-produced materials were perceived as highly acceptable, culturally resonant, and were associated with strong reported intentions to act. The inclusion of persons with disabilities, further enhance the inclusivity and accessibility of the messaging for example, participants emphasized the use of clear multimodal formats (visual, audio, local language) to ensure that everyone could engage with the messages. Also, the inclusion and attempt to improve accessibility for minorities, also improves utility and accessibility for majorities. These outcomes challenge the dominance of top-down, expert-led AMR campaigns and point toward a more equitable model of health communication rooted in participatory action. Importantly, the findings indicate that such co-produced interventions are more likely to be perceived as effective, relatable, and actionable. However, realizing the full potential of this model requires addressing key structural barriers such as limited healthcare access, unregulated antibiotic markets, and inadequate policy enforcement. In this regard, co-production should not be seen as a substitute for system-wide reform but rather as a complementary strategy that grounds public health messages in local realities. Future studies should explore the long-term impact of such interventions on actual antibiotic use behaviours and investigate how co-production can be scaled in resource-constrained settings without losing contextual sensitivity. Finally, this study contributes to a growing body of evidence advocating for participatory, grassroots-driven solutions in global health, emphasizing that sustainable behaviour change is more likely when communities are treated not just as recipients of information but as co-creators of knowledge and action.

## Supporting information

S1 Text Pre & Post Survey Copy. (PDF)

S1 Checklist Inclusivity in Global Research. (PDF)

S1 Table Full Workshop Participant Demographics. (PDF)

S2 Table Summary of pre and post survey response distributions. (PDF)

## References

[1] Ho CS, Wong CTH, Aung TT, Lakshminarayanan R, Mehta JS, Rauz S, et al. Antimicrobial resistance: a concise update. Lancet Microbe. 2025;6(1):100947. doi: 10.1016/j.lanmic.2024.07.010 39305919

[2] Janssen J, Afari-Asiedu S, Monnier A, Abdulai MA, Tawiah T, Wertheim H, et al. Exploring the economic impact of inappropriate antibiotic use: the case of upper respiratory tract infections in Ghana. Antimicrob Resist Infect Control. 2022;11(1):53. doi: 10.1186/s13756-022-01096-w 35365210 PMC8973739

[3] Murray CJ, Ikuta KS, Sharara F, Swetschinski L, Aguilar GR, Gray A, et al. Global burden of bacterial antimicrobial resistance in 2019: a systematic analysis. The Lancet. 2022;399(10325):629–55. 10.1016/S0140-6736(21)02724-0 PMC8841637 35065702

[4] Walsh TR, Gales AC, Laxminarayan R, Dodd PC. Antimicrobial Resistance: Addressing a Global Threat to Humanity. PLoS Med. 2023;20(7):e1004264. doi: 10.1371/journal.pmed.1004264 37399216 PMC10317217

[5] Otaigbe II, Elikwu CJ. Drivers of inappropriate antibiotic use in low- and middle-income countries. JAC Antimicrob Resist. 2023;5(3):dlad062. doi: 10.1093/jacamr/dlad062 37265987 PMC10230568

[6] Dar OA, Hasan R, Schlundt J, Harbarth S, Caleo G, Dar FK, et al. Exploring the evidence base for national and regional policy interventions to combat resistance. The Lancet. 2016;387(10015):285–95. doi: 10.1016/s0140-6736(15)00520-6 26603921

[7] Sharma A, Singh A, Dar MA, Kaur RJ, Charan J, Iskandar K, et al. Menace of antimicrobial resistance in LMICs: Current surveillance practices and control measures to tackle hostility. J Infect Public Health. 2022;15(2):172–81. doi: 10.1016/j.jiph.2021.12.008 34972026

[8] Olamijuwon E, Keenan K, Mushi MF, Kansiime C, Konje ET, Kesby M, et al. Treatment seeking and antibiotic use for urinary tract infection symptoms in the time of COVID-19 in Tanzania and Uganda. J Glob Health. 2024;14:05007. doi: 10.7189/jogh.14.05007 38236690 PMC10795859

[9] Cabral C, Zhang T, Oliver I, Little P, Yardley L, Lambert H. Influences on use of antibiotics without prescription by the public in low- and middle-income countries: a systematic review and synthesis of qualitative evidence. JAC Antimicrob Resist. 2024;6(5):dlae165. doi: 10.1093/jacamr/dlae165 39464857 PMC11503652

[10] Olamijuwon E, Konje E, Kansiime C, Kesby M, Keenan K, Neema S, et al. Antibiotic dispensing practices during COVID-19 and implications for antimicrobial resistance (AMR): parallel mystery client studies in Uganda and Tanzania. Antimicrob Resist Infect Control. 2023;12(1):10. doi: 10.1186/s13756-022-01199-4 36774512 PMC9919751

[11] Chandler CIR. Current accounts of antimicrobial resistance: stabilisation, individualisation and antibiotics as infrastructure. Palgrave Commun. 2019;5(1):53. doi: 10.1057/s41599-019-0263-4 31157116 PMC6542671

[12] Bergsholm YKR, Feiring M, Charnock C, Holm LB, Krogstad T. Exploring patients’ adherence to antibiotics by understanding their health knowledge and relational communication in encounters with pharmacists and physicians. Explor Res Clin Soc Pharm. 2023;12:100372. doi: 10.1016/j.rcsop.2023.100372 38089697 PMC10711180

[13] Llor C, Bjerrum L. Antimicrobial resistance: risk associated with antibiotic overuse and initiatives to reduce the problem. Ther Adv Drug Saf. 2014;5(6):229–41. doi: 10.1177/2042098614554919 25436105 PMC4232501

[14] Ngoh LN, Shepherd MD. Design, development, and evaluation of visual aids for communicating prescription drug instructions to nonliterate patients in rural Cameroon. Patient Educ Couns. 1997;31(3):245–61. doi: 10.1016/s0738-3991(97)89866-7 9277247

[15] Haldane V, Chuah FLH, Srivastava A, Singh SR, Koh GCH, Seng CK, et al. Community participation in health services development, implementation, and evaluation: A systematic review of empowerment, health, community, and process outcomes. PLoS One. 2019;14(5):e0216112. doi: 10.1371/journal.pone.0216112 31075120 PMC6510456

[16] Rustage K, Crawshaw A, Majeed-Hajaj S, Deal A, Nellums L, Ciftci Y, et al. Participatory approaches in the development of health interventions for migrants: a systematic review. BMJ Open. 2021;11(10):e053678. doi: 10.1136/bmjopen-2021-053678 34697122 PMC8548676

[17] Neuhauser L, Rothschild B, Graham C, Ivey SL, Konishi S. Participatory design of mass health communication in three languages for seniors and people with disabilities on Medicaid. Am J Public Health. 2009;99(12):2188–95. doi: 10.2105/AJPH.2008.155648 19833990 PMC2775764

[18] Villar ME, Johnson PW. Tailoring Content for Authenticity and Adoption: Community-Based Participatory Research and the Co-creation of Story-Based Health Communication for Underserved Communities. Front Commun. 2021;6. doi: 10.3389/fcomm.2021.663389

[19] Greenhalgh T, Jackson C, Shaw S, Janamian T. Achieving Research Impact Through Co-creation in Community-Based Health Services: Literature Review and Case Study. Milbank Q. 2016;94(2):392–429. doi: 10.1111/1468-0009.12197 27265562 PMC4911728

[20] Chukwu EE, Oladele DA, Awoderu OB, Afocha EE, Lawal RG, Abdus-Salam I, et al. A national survey of public awareness of antimicrobial resistance in Nigeria. Antimicrob Resist Infect Control. 2020;9(1):72. doi: 10.1186/s13756-020-00739-0 32434552 PMC7238560

[21] Nigeria Centre for Disease Control and Prevention. National Action Plan on Antimicrobial Resistance: Situational Analysis and Key Recommendations for the Development of NAP 2.0. 2024. Available at: https://ncdc.gov.ng/themes/common/files/establishment/7ba06feaab1fd7a197ff9c05b6140225.pdf

[22] Alabi ED, Rabiu AG, Adesoji AT. A review of antimicrobial resistance challenges in Nigeria: The need for a one health approach. One Health. 2025;20:101053. doi: 10.1016/j.onehlt.2025.101053 40370425 PMC12077226

[23] Esther J, Onyebuchi OB, Eugenia OE, Ann OC, Adamma NM, Chiaka IG, et al. Antimicrobial resistance in Nigeria’s healthcare system: a comprehensive narrative review and policy implications. Discov Public Health. 2025;22(1). doi: 10.1186/s12982-025-00859-1

[24] World Health Organization. Nigeria Launches First National Antimicrobial Resistance Survey. 2025. Available at: https://www.afro.who.int/countries/nigeria/news/nigeria-launches-first-national-antimicrobial-resistance-survey#:~:text=Nigeria%20ranks%2020th%20globally%20for,%E2%80%A2

[25] Lara A, 2025. FG to tackle drug resistance, reactivate national antimicrobial network. Available at: https://punchng.com/fg-to-tackle-drug-resistance-reactivate-national-antimicrobial-network/

[26] Maduko W, Olamijuwon E, Kesby M, Hale JM. Public-targeted interventions addressing antimicrobial resistance and antibiotic use in Sub-Saharan Africa: a scoping review. BMJ Glob Health. 2025;10(3):e017455. doi: 10.1136/bmjgh-2024-017455 40118465 PMC11931924

[27] Ghiga I, Sidorchuk A, Pitchforth E, Stålsby Lundborg C, Machowska A. ’If you want to go far, go together’-community-based behaviour change interventions to improve antibiotic use: a systematic review of quantitative and qualitative evidence. J Antimicrob Chemother. 2023;78(6):1344–53. doi: 10.1093/jac/dkad128 37147849 PMC10232266

[28] Ledingham K, Hinchliffe S, Jackson M, Thomas F, Tomson G. Antibiotic resistance: using a cultural contexts of health approach to address a global health challenge. World Health Organization; 2019.

[29] National Population Commission Nigeria NPC. Nigeria Population Projection and Demographic Indicators-State and National. Abuja, Nigeria: National Population Commission; 2020.

[30] National Population Commission. Nigeria Demographic and Health Survey Summary Report 2024. 2024. Available at: https://dhsprogram.com/pubs/pdf/SR294/SR294.pdf

[31] Kesby M, Fredricks K. Participatory design of public health messages on antibiotic use and AMR. 2022. doi: 10.5281/zenodo.7319094

[32] Kindon S, Pain R, Kesby M. Critically Engaging Participatory Action Research. Critically Engaging Participatory Action Research. Routledge. 2024. p. 1–29. doi: 10.4324/9780429400346-1

[33] Vaughn LM, Jacquez F. Participatory Research Methods – Choice Points in the Research Process. Journal of Participatory Research Methods. 2020;1(1). doi: 10.35844/001c.13244

[34] Kindon S, Pain R, Kesby M. Participatory action research: Origins, approaches and methods. Participatory action research approaches and methods. Routledge; 2007. p. 35–44.

[35] Skinner CS, Tiro J, Champion VL. Background on the health belief model. Health behavior: Theory, research, and practice. 2015;75:1–34.

[36] Champion VL, Skinner CS. The health belief model. Health behavior and health education: Theory, research, and practice. 2008. p. 45–65.

[37] Alyafei A, Easton-Carr R. The Health Belief Model of Behavior Change. StatPearls. Treasure Island (FL): StatPearls Publishing; 2024. 39163427

[38] Denzin NK, Lincoln YS. The Sage Handbook of Qualitative Research. Sage; 2011.

[39] Braun V, Clarke V. Thematic analysis: A practical guide. 2021.

[40] Freire K, Pope R, Jeffrey K, Andrews K, Nott M, Bowman T. Engaging with Children and Adolescents: A Systematic Review of Participatory Methods and Approaches in Research Informing the Development of Health Resources and Interventions. Adolescent Res Rev. 2022;7(3):335–54. doi: 10.1007/s40894-022-00181-w

[41] Rogers EM, Singhal A, Quinlan MM. Diffusion of innovations. An integrated approach to communication theory and research. Routledge; 2014. p. 432–48.

[42] Gréaux M, Moro MF, Kamenov K, Russell AM, Barrett D, Cieza A. Health equity for persons with disabilities: a global scoping review on barriers and interventions in healthcare services. Int J Equity Health. 2023;22(1):236. doi: 10.1186/s12939-023-02035-w 37957602 PMC10644565

[43] Hastings G, Stead M, Webb J. Fear appeals in social marketing: Strategic and ethical reasons for concern. Psychology and Marketing. 2004;21(11):961–86. doi: 10.1002/mar.20043

[44] Witte K, Allen M. A meta-analysis of fear appeals: implications for effective public health campaigns. Health Educ Behav. 2000;27(5):591–615. doi: 10.1177/109019810002700506 11009129

