# Supplementary material for: Engaging communities in addressing antimicrobial resistance: Co-producing locally relevant public health messages
Source: PLOS Glob Public Health. 2026 Apr 17;6(4):e0006212. doi: 10.1371/journal.pgph.0006212 (PMC13089702; doi:10.1371/journal.pgph.0006212)
Supplement: S1 Text — (PDF) [file pgph.0006212.s001.pdf]

Public Engagement Event Pre-Survey: Co-Produced AMR Public Health Messaging Materials

Thank you for participating in this public engagement session. We are interested in understanding your initial thoughts and perceptions of the co-produced public health messages on antimicrobial resistance (AMR) and antibiotic stewardship. Please answer the following questions honestly. Your feedback will help us evaluate the acceptability and effectiveness of these materials.

Demographic Information:

1. **Age:** (a) 18–25 (b) 26–35 (c) 36–45 (d) 46–55 (e) 56 and above
2. **Gender:** [ ] Male [ ] Female [ ] Other (please specify) __________
3. **Education Level:** [ ] Primary [ ] Secondary [ ] Tertiary [ ] Other (please specify) _________
4. **Do you have access to:** (a) TV (b) Radio (c) Mobile phone (specify type: basic or smartphone) (d) Internet/social media (e.g., WhatsApp, TikTok, Facebook, etc.)
5. **Have you used antibiotics recently?** (a) In the last week (b) In the last month (c) In the last six months (d) More than six months ago (e) Never
6. **Do you consider yourself a frequent user of antibiotics?** (a) Yes (b) No (c)Occasionally
7. **Have you ever obtained antibiotics to treat yourself, family members or others without medical advice?** (a) Yes (b) No (c) Occasionally

Awareness of Antimicrobial Resistance (AMR):

8. **How aware are you of antimicrobial resistance (AMR)?** (a) Not at all aware (b)Somewhat aware (c) Moderately aware (d) Very aware
9. **Before today, have you seen or heard any public health messages about AMR or antibiotic use?** (a) Yes (b) No
10. **If yes, please write where:**
11. **How likely do you think people in your community would pay attention to public health messages about AMR or antibiotic use?** (a) Very unlikely(b) Unlikely (c) Likely (d) Very likely

Initial Perceptions:

10. **Before seeing the co-produced materials, how important do you think it is to educate the public about AMR and responsible antibiotic use?**
    (a) Not important (b) Slightly important (c) Moderately important (d) Very important

Expectations:

13. **In general terms, what kinds of public health information are most likely to prove effective?** [ ] Those produced by experts [ ] Those produced by the public [ ] Those co-produced by experts and the public.

14. **What makes the best public health campaigns effective in your opinion:** __________

15. **How effective do you expect the co-produced materials (posters, jingles, short film) to be in raising awareness about antimicrobial resistance (AMR)?**
    (a) Not effective (b) Slightly effective (c) Moderately effective (d) Very effective (e) Extremely effective.

16. **Do you think the involvement of community members in creating these materials will increase their effectiveness?** (a) Strongly disagree (b) Disagree
    (c) Neutral (d) Agree (e) Strongly agree

Public Engagement Event Post-Survey: Co-Produced AMR Public Health Messaging Materials

Effectiveness of Co-Produced Materials:

1. **After viewing/experiencing the materials, how effective do you think they are in raising your awareness and public awareness about AMR?** (a) Not effective (b) Slightly effective (c) Moderately effective (d) Very effective (e) Extremely effective
2. **Which of the co-produced materials did you find most impactful, and why?** (a) Posters (b) Jingles (c) Short film (d) All were equally impactful
   Please explain your choice:
3. **Which medium would best be used to disseminate these materials in your community (e.g., WhatsApp, TikTok, radio, TV)?**
   (a) Radio (b) TV (c) social media (please specify platform) _________
   (d) Other _________

Acceptability and Cultural Relevance:

4. **To what extent do you agree that the co-produced materials were culturally appropriate for your community?**
   (a) Strongly disagree (b) Disagree (c) Neutral (d) Agree (e) Strongly agree
5. **What makes the materials culturally appropriate or not?** ____________
6. **How comfortable were you with the language, imagery, and overall presentation of the materials?** (a)Not comfortable at all (b)Slightly comfortable (c)Moderately comfortable (d)Very comfortable (e)Extremely comfortable

Behavioural Intentions and Engagement:

7. **After this session, how likely are you to take specific actions to prevent AMR (e.g., using antibiotics responsibly, spreading awareness)?**
   (a) Not likely at all (b) Slightly likely (c) Moderately likely (d) Very likely (e) Extremely likely
   What specific actions do you plan to take? ________
8. **Would you recommend these materials be used in other communities to raise awareness about AMR?** (a)Definitely not (b)Probably not (c)Not sure (d)Probably yes (e)Definitely yes
9. **What was the most useful thing you learned from the materials?**
10. **Do you think involving more community members in the creation of such materials could improve their effectiveness?** (a)Strongly disagree (b)Disagree (c)Neutral (d)Agree (e)Strongly agree
11. **How do you think these materials could be improved for greater effectiveness?**
