# Supplementary material for: Engaging communities in addressing antimicrobial resistance: Co-producing locally relevant public health messages
Source: PLOS Glob Public Health. 2026 Apr 17;6(4):e0006212. doi: 10.1371/journal.pgph.0006212 (PMC13089702; doi:10.1371/journal.pgph.0006212)
Supplement: S1 Table — (PDF) [file pgph.0006212.s003.pdf]

***S1 Table: Full Table Showing** Demographic characteristics distribution of co-production workshop participants (n=30), PWD: Persons with disabilities*

**Participant**	**Gender**	**PWD**	**Age Group**	**Education**
P1	F	No	18–30	Tertiary
P2	F	No	18–30	Tertiary
P3	F	No	18–30	Tertiary
P4	F	No	18–30	Tertiary
P5	M	No	18–30	Tertiary (Still in school)
P6	M	No	18–30	Tertiary (Still in school)
P7	M	Yes	31–50	No formal education
P8	M	Yes	31–50	Primary
P9	M	Yes	31–50	Primary
P10	M	Yes	31–50	Secondary
P11	M	No	31–50	Secondary
P12	M	No	31–50	Secondary
P13	M	No	31–50	Secondary
P14	M	No	31–50	Tertiary
P15	M	No	31–50	Tertiary
P16	M	No	31–50	Tertiary
P17	M	No	31–50	Tertiary
P18	F	No	31–50	Tertiary
P19	F	No	31–50	Tertiary
P20	F	No	31–50	Tertiary
P21	F	No	31–50	Tertiary
P22	F	No	31–50	Secondary
P23	F	No	31–50	Primary
P24	F	No	31–50	Primary
P25	F	No	31–50	Primary
P26	F	No	31–50	Secondary
P27	F	No	31–50	Secondary
P28	M	Yes	51+	No formal education
P29	F	No	51+	Secondary
P30	F	No	51+	Tertiary
