# Supplementary material for: Engaging communities in addressing antimicrobial resistance: Co-producing locally relevant public health messages
Source: PLOS Glob Public Health. 2026 Apr 17;6(4):e0006212. doi: 10.1371/journal.pgph.0006212 (PMC13089702; doi:10.1371/journal.pgph.0006212)
Supplement: S2 Table — (PDF) [file pgph.0006212.s004.pdf]

S2 Table: Summary of pre and post survey response distributions

Category	Subcategory	Number (n)	Percentage (%)
**Have you used antibiotics recently?**	Other/unclear	1	2
	Never	2	4
	In the last week	4	8
	In the last month	7	14
	In the last six months	14	28
	More than six months ago	22	44
**Do you consider yourself a frequent user of antibiotics?**	Yes	5	10
	Occasionally	15	30
	No	30	60
**Have you ever obtained antibiotics to treat yourself, family members or others without medical advice?**	Occasionally	6	12
	No	11	22
	Yes	33	66
**Before seeing the co-produced materials, how important do you think it is to educate the public about AMR and responsible antibiotic use?**	Slightly important	2	4
	Moderately important	5	10
	Very important	43	86
**In general terms, what kinds of public health information are most likely to prove effective?**	Other	1	2
	Produced by public	3	6
	Produced by experts	15	30
	Co-produced by experts and public	31	62
**What are your expectations for the effectiveness of the co-produced materials (posters, jingles, short film) in raising awareness about AMR?**	Not effective	1	2
	Slightly effective	4	8
	Moderately effective	9	18

	Extremely effective	15	30
	Very effective	21	42

**Do you (agree) think the involvement of community members in creating these materials will increase their effectiveness?**	Strongly disagree	1	2
	Neutral	1	2
	Disagree: 2 respondents	2	4
	Agree	12	24
	Strongly agree	35	68
**After viewing/experiencing the materials, how effective do you think they are in raising your awareness and public awareness about AMR?**	Not effective	0	0
	Slightly Effective	1	2
	Moderately Effective	3	6
	Very Effective	23	46
	Extremely Effective	23	46
**Which of the co-produced materials did you find most impactful land why? ***	Identified posters or a combination of posters and jingles as the most impactful	3	6
	Found jingles most impactful	5	10
	Found the short film to be the most impactful.	11	22
	Indicated that all the co-produced materials (posters, jingles, and short film) were equally impactful.	30	60
**To what extent do you agree that the coproduced materials were culturally appropriate for your community?**	Neutral	6	
	Agree	11	
	Strongly Agree	33	
**Do you think involving more community members in the creation of such materials could improve their effectiveness?**	Disagree	2	4
	Agree	9	18
	Strongly Agree	39	78

**How comfortable were you with the language, imagery, and overall presentation of the materials?**	Slightly comfortable	1	
	Moderately comfortable	7	
	Extremely comfortable	20	
	Very comfortable	23	
**After this session, how likely are you to take specific actions to prevent AMR (e.g.**	Moderately likely	4	8
**using antibiotics responsibly, spreading awareness)?**			
	Extremely likely	22	44
	Very likely	24	48
**Would you recommend these materials be used in your community and other communities to raise awareness about AMR?**	Probably yes	9	18
	Yes	41	82

***Please explain your choice (why) for the question (Which of the co-produced materials did you find most impactful)? Identified Themes & Frequency:**

**Theme**	**Description**	**Count**
**Multi-format impact**	Materials were effective because they included visuals, audio, text, etc.	6
**Short film as memorable & relatable**	Film is engaging, relatable, and leaves a lasting impression	4
**Inclusive communication (accessible to all)**	Materials were appreciated for being understandable by both literate and non-literate audiences	3
**Use of local language and cultural relevance**	Accessibility through local language, making it easier for uneducated members to understand	2
**Jingles as effective reminders**	Audio-based messages like jingles helped reinforce the message	2
**Engaging and interesting content**	Comments on how the content captured attention and was easy to follow	2
**Information complementarity**	Different formats covered different things (posters, film, jingle each contributed uniquely)	2

Sample Quotes by Theme:

- **Multi-format impact**:
  "They all stimulated the various means of receiving information, visual and audio with captivating pictures and colours."
- **Short film is memorable**:
  "Because short film is been seen with our eyes which makes it easier to remember and also we can see the dangerous effect of drug abuse/AMR."
- **Inclusive communication**:

"They were all equally impactful because whether you are dumb, deaf or blind you still have a chance to be informed."

- **Local language**:
  "The message is been spoken in a way [local language] that uneducated can understand."
- **Jingles as reminders**:
  "It is the jingle that makes the produced materials very effective."
- **Engaging content**:
  "It was interesting and engaging because it was both visual and audio and involved public contribution."
- **Complementarity**:
  "There was something the poster said that the jingles did not and some the film said that the poster did not say."
